# Effect of *Gracilaria vermiculophylla* Macroalga on Non-Alcoholic Fatty Liver Disease in Obese Rats

**DOI:** 10.3390/antiox13030369

**Published:** 2024-03-18

**Authors:** Maitane González-Arceo, Leixuri Aguirre, María Teresa Macarulla, Clàudia Gil-Pitarch, María Luz Martínez-Chantar, María P. Portillo, Saioa Gómez-Zorita

**Affiliations:** 1 Nutrition and Obesity Group, Department of Pharmacy and Food Science, Faculty of Pharmacy and Lucio Lascaray Research Centre, University of the Basque Country (UPV/EHU), 01006 Vitoria-Gasteiz, Spain; maitane.gonzalez@ehu.eus (M.G.-A.); mariateresa.macarulla@ehu.eus (M.T.M.); mariapuy.portillo@ehu.eus (M.P.P.); saioa.gomez@ehu.eus (S.G.-Z.); 2 CIBERobn Physiopathology of Obesity and Nutrition, National Institute of Health Carlos III, 28222 Madrid, Spain; 3 BIOARABA Health Research Institute, 01006 Vitoria-Gasteiz, Spain; 4 Liver Disease Lab, Center for Cooperative Research in Biosciences (CIC bioGUNE), Basque Research and Technology Alliance (BRTA), 48160 Derio, Spain; cgil@cicbiogune.es (C.G.-P.); mlmartinez@cicbiogune.es (M.L.M.-C.); 5 Centro de Investigación Biomédica en Red de Enfermedades Hepáticas y Digestivas (CIBERehd), National Institute of Health Carlos III, 28222 Madrid, Spain

**Keywords:** alga, *Gracilaria vermiculophylla*, non-alcoholic fatty liver disease, oxidative stress, Zucker rat

## Abstract

Marine algae are valuable sources of bioactive compounds that have the potential to be used in the management of various pathologies. Despite the increasing prevalence of NAFLD, the absence of an approved effective pharmacological treatment with demonstrable effectiveness persists. In this context, the aim of the present study is to assess the effect of *Gracilaria vermiculophylla* red seaweed dietary supplementation on hepatic lipid accumulation, as well as on oxidative stress, inflammation and fibrosis- related markers on obese *fa*/*fa* Zucker rats fed with a standard diet, supplemented or not with 2.5% or 5% dehydrated *Gracilaria vermiculophylla*. After a six-week supplementation with the macroalga, no significant reduction in hepatic total lipid content or hepatic triglyceride content was observed. However, both doses were able to diminish hepatic NEFA concentration by reducing de novo lipogenesis and increasing mitochondrial biogenesis. Moreover, supplementation with the dose of 2.5% improved some oxidative stress and inflammation-related markers. Supplementation with the dose of 5% did not exert these clear beneficial effects. Thus, this study demonstrates that while *Gracilaria vermiculophylla* may not mitigate hepatic steatosis, it could exert protective effects on the liver by reducing NEFA content and enhancing oxidative stress and inflammation parameters.

## 1. Introduction

Non-alcoholic fatty liver disease (NAFLD) currently stands as a predominant contributor to chronic liver diseases worldwide, with an estimated prevalence of approximately 30% [1]. Recently, it was renamed a metabolic (dysfunction)-associated fatty liver disease (MAFLD) in order to emphasise the underlying metabolic dysfunction [2,3]. However, owing to the lack of consensus among all expert committees, the term NAFLD is used throughout the present manuscript.

NAFLD encompasses a variety of hepatic dysfunctions, ranging from simple steatosis to non-alcoholic steatohepatitis (NASH), which may present with or without fibrosis and can progress to cirrhosis and hepatocellular carcinoma. Simple steatosis is characterised by an increased accumulation of lipids within the hepatocytes, exceeding 5% of liver weight. In NASH, this lipid accumulation is accompanied by inflammation and hepatocyte injury [4]. NAFLD is strongly associated with insulin resistance, which causes an imbalance in lipid metabolism in favour of hepatic lipid accrual. The excessive accumulation of lipid content causes hepatocyte damage. Specifically, elevated levels of non-esterified fatty acids (NEFAs) can give rise to toxic lipid species, such as diacylglycerols, ceramides or lysophosphatidylcholine. This results in lipotoxicity, which triggers oxidative stress and the production of reactive oxygen species (ROS) [5,6]. Increased oxidative stress plays a crucial role in the progression of NAFLD to NASH [7] because it damages cellular structures and function, causing hepatocyte injury and death [8]. Presently, there exists no approved effective pharmacological treatment. Consequently, the scientific community is in pursuit of novel sources of molecules with potential utility in the prevention and treatment of NAFLD.

Marine macroalgae or seaweeds are considered a viable and sustainable source of both micro- and macronutrients that increase the nutritional value of the diet [9]. Furthermore, they have gained a lot of interest due to their numerous and unique bioactive compounds, not present in terrestrial food sources. These include characteristic pigments, phenolic compounds, polyunsaturated fatty acids, polysaccharides or peptides [10]. It is scientifically proven that these compounds exhibit the potential to improve human health through their documented antioxidant, anti-inflammatory, anticancer, antidiabetic, antihypertensive, antihyperlipidemic and antiobesity effects [9,11].

The red macroalga *Gracilaria* sp. is predominantly utilised for agar production and as a source of sulphated polysaccharides in the pharmaceutical and biotechnology sectors [12]. Moreover, studies have described the potential of *Gracilaria* sp. to modulate glucose, cholesterol and lipid metabolism [13,14,15,16]. In fact, in a preceding study conducted by our group, it was observed that an extract derived from *Gracilaria vermiculophylla*, particularly rich in proteins and peptides, demonstrated the ability to reduce triglyceride accumulation in cultured hepatocytes by increasing fatty acid oxidation [17]. Nevertheless, there are no studies investigating the impact of dietary supplementation with *Gracilaria vermiculophylla* on in vivo models.

In this context, the aim of this study is to assess the influence of long-term dietary supplementation with *Gracilaria vermiculophylla*, a red macroalga, on hepatic lipid accumulation in Zucker obese (*fa*/*fa*) rats, a genetic model of steatosis. Additionally, the study aims to elucidate the potential underlying mechanisms involved. Furthermore, other parameters related with the early progression to NASH, such us oxidative stress and inflammation, are also assessed.

## 2. Materials and Methods

### 2.1. Macroalga Obtention and Characterisation

*Gracilaria vermiculophylla* specimens were harvested from the northwest Iberian coast and subsequently dehydrated by Porto Muiños S.L. (Cambre, Spain). *Gracilaria vermiculophylla* is a cartilaginous red macroalga (phyllum *Rhodophyta*) that arises from a discoid holdfast with a densely and irregularly branched thallus. Branches are cylindrical and slightly constricted at the base. The colour varies from dark red to reddish brown, occasionally appearing greenish or black, a characteristic that differentiates it from other species of the same genus such as *Gracilaria gracilis*, which has an intense red colour. It is mainly found in estuaries and intertidal habitats, often in shallow waters in sheltered areas influenced by freshwater. *Gracilaria vermiculophylla* tolerates broad ranges of temperature, light, salinity and nutrient levels. It usually grows unattached in loose-lying mats on mud and sand, and it is generally found in vegetative state with few reproductive structures [18,19].

Composition analysis was carried out by Corporación Laber S.L. (Santiago de Compostela, Spain). Briefly, total lipids were determined by the Weibull method, a variation of the Soxhlet method where the sample is subjected to hydrochloric acid digestion prior to lipid extraction. Saturated fatty acids were assessed by gas chromatography (GC) of fatty acid methyl esters (FAMEs). Protein content was determined by measuring the total nitrogen content using the Kjeldahl method. Simple carbohydrates were quantified by high-performance liquid chromatography with refractive index detector (HPLC-RI). Fibre content was assessed by an enzymatic–gravimetric method, which involved treating the sample with amylase, protease and amyloglucosidase. Ashes were examined by burning off the organic matter by heating the sample at 550 °C overnight to in a muffle furnace. Moisture of the samples was analysed through a drying process at 105 °C to a constant weight. Lastly, the carbohydrate content was calculated by the difference in 100 g of dehydrated macroalga. In the case of total polyphenols, these were quantified following the Folin–Ciocalteu method.

### 2.2. Animals, Diets and Experimental Design

The study was conducted on thirty-six male, homozygous, obese (*fa*/*fa*) Zucker rats and nine male lean (*Fa*/*?*) Zucker rats, aged eight weeks, purchased from Charles River Laboratories (Lyon, France). Animals were housed in polycarbonate cages (two rats per cage), placed in an air-conditioned room (22 ± 2 °C) with a 12 h light/dark cycle, and fed with a standard diet (2014 Global Diet, Envigo, Udine, Italy) containing 4% lipids, 14.5% proteins and 48% carbohydrates and providing 2.9 kcal/g. After a 6-day adaptation period, the obese and lean rats were assigned to different experimental groups (n = 9/group). One group of obese rats was fed solely the standard diet (OC group), while two additional groups received the standard diet enriched with either 2.5% or 5% dehydrated *Gracilaria vermiculophylla* (LGV and HGV groups, respectively). The dehydrated macroalga was ground to powder using a blender, and then this powder was mixed daily with the standard diet until the mixture was homogeneous. Experimental groups that did not receive that macroalga were also provided with the same standard diet. Lean rats were assigned to the lean control group (LC) to function as the healthy control cohort in the study. Due to the fact that when compared to OC group, the HGV group significantly decreased its food intake shortly after the initiation of the experiment, a pair-fed (PF) group was introduced. The PF group received an amount of the standard diet equivalent to that consumed by rats in the HGV group on the preceding day. Water and food were provided ad libitum throughout the 6-week experimental period (long-term treatment), with the exception of the pair-fed group. Daily records of body weight and food intake were maintained. Additionally, the Energy Efficiency Ratio (EER) (body weight gain expressed in grams/energy intake expressed in kcal) was calculated.

Upon completion of the experimental period and subsequent to a 12 h fasting interval, blood samples were collected from the tail for glucose and insulin determinations. The animals were then euthanised through cardiac exsanguination under anaesthesia (chloral hydrate). The liver was weighed and promptly frozen. A section of the larger liver lobule from each animal was reserved for Oil Red O staining. Serum was extracted from blood samples following centrifugation (1000× *g* for 10 min, 4 °C). All samples were stored at −80 °C until analysis. The experiment adhered to the institution’s guidelines for the care and use of laboratory animals (M20_2021_214).

### 2.3. Serum Parameters

Serum parameters were analysed using commercially available kits: triglycerides (1001313, Spinreact, Girona, Spain), free glycerol (F6428, Sigma, Saint Louis, MO, USA), aspartate aminotransferase (AST/GOT) (1001161, Spinreact, Girona, Spain), alanine aminotransferase (ALT/GPT) (1001171, Spinreact, Girona, Spain), alkaline phosphatase (ALP) (1001131, Spinreact, Girona, Spain), NEFAs (434-91795, Fujifilm, Neuss, Germany), glucose (11504, BioSystems, Barcelona, Spain), insulin (EZRMI-13K, Millipore, Darmstadt, Germany) and uric acid (41000, Spinreact, Girona, Spain). Triglyceride content was calculated taking into account the amount of free glycerol.

Insulin sensitivity was evaluated using two indices: the homeostatic model assessment for insulin resistance (HOMA-IR) and the revised quantitative insulin sensitivity check index (R-QUICKI). The HOMA-IR was calculated from basal insulin and glucose values using Matthews’ formula [20]:

HOMA-IR = (fasting glucose [mmol/L] × fasting insulin [mU/L])/22.5

R-QUICKI was calculated using fasting glucose, insulin and NEFA concentrations [21]:

R-QUICKI = 1/(log fasting glucose [mg/dL] + fasting insulin [μU/mL] + fasting NEFAs [mmol/L])

### 2.4. Hepatic Lipid Content

The lipid content in liver samples was assessed by analysing tissue sections embedded in the OCT embedding Matrix for Frozen Sections (Pioneer, PRC/OCT). These sections, cut into 5 µm thick samples using a Leica CM1850 Cryostat, were subsequently fixed in a 10% Formalin solution (neutral buffer, Sigma-Aldrich, HT501128) for 2 min. Following fixation, the slices were treated with 60% isopropanol and stained using freshly prepared oil red (Sigma-Aldrich, O0625-25g). After a 2 min wash with 60% isopropanol, the samples were counterstained with Mayer’s Hematoxylin (Sigma MHS32) and then mounted with an aqueous mounting medium [22]. No alterations were made to the histological microscopy images.

Moreover, liver samples weighing 100–200 mg were homogenised in 2 mL of buffer containing 10 mM Tris-HCl, 2 mM ethylenediaminetetraacetic acid (EDTA), and 250 mM sucrose (pH = 7.4). Commercially available spectrophotometric kits were used to measure hepatic triglyceride (1001313, Spinreact, Girona, Spain) and NEFA (434-91795, Fujifilm, Neuss, Germany) contents. Free glycerol (F6428, Sigma, Saint Louis, USA) was also quantified in order to perform free glycerol blanking for triglyceride measurements.

### 2.5. Enzyme Activities in Liver

Regarding the analysis of lipogenic enzymes, fatty acid synthase (FAS) activity was determined spectrophotometrically using the method outlined by Lynen [23] in the cytosolic fraction of liver homogenates. Briefly, liver samples (200–300 mg) were homogenised in 2 mL buffer (pH 7.4) containing 250 mM sucrose, 1 mM EDTA and 10 mM Tris-HCl, and then centrifugated (700× *g*, 10 min, 4 °C). The supernatant was collected and subjected to a second centrifugation step (12,000× *g*, 15 min, 4 °C). Following the second centrifugation, the supernatant was used to determine FAS activity from the rate of malonyl-CoA dependent nicotinamide adenine dinucleotide phosphate (NADPH) oxidation. The results were expressed as consumed NADPH nmol·min^−1^·mg^−1^ protein.

With regard to fatty acid oxidation, the activities of carnitine palmitoyltransferase-1a (CPT-1a) and acyl-CoA oxidase (ACO) were measured in the mitochondrial/peroxisomal fraction of liver homogenates using the methods described by Bieber [24] and Lazarow [25], respectively. After the second centrifugation, the resulting pellet was re-suspended in resuspension buffer (pH 7.4) comprising sucrose (70 mM), mannitol (200 mM), 4-(2-hydroxyethyl)-1-piperazineethanesulfonic acid (HEPES) (2 mM) and EDTA (1 mM). CPT-1a activity was expressed as released CoA nmol·min^−1^ mg^−1^ protein, and ACO activity as NADH nmol formed·min^−1^·mg^−1^ protein.

Concerning hepatic triglyceride release, microsomal triglyceride transfer protein (MTP) activity was assessed in liver samples obtained by homogenising 100 mg of liver in 1 mL of homogenisation buffer, which contained Tris-HCl (10 mM), NaCl (150 mM) and EDTA (1 mM) at pH 7.4. The homogenate was then centrifuged (7500× *g*, 30 min, 4 °C). MTP activity was measured fluorometrically in the supernatant by means of a commercial kit (MTP Activity Assay Kit, MAK 110, Sigma-Aldrich, St. Louis, USA) and expressed as a percentage of transference·h^−1^·mg^−1^ protein.

In all instances, total protein content from samples was quantified using the Bradford method [26], with bovine serum albumin (BSA) serving as the standard.

### 2.6. Western Blotting for Protein Expression Measurement

Diacylglycerol acyltransferase 2 (DGAT2), fatty acid transport protein 2 (FATP2), acetyl-CoA carboxylase (ACC), phosphorylated acetyl-CoA carboxylase (ACC), mitochondrial transcription factor A (TFAM), aquaglyceroporin 9 (AQP9), FAS, carbohydrate responsive element binding protein (CHREBP), nuclear respiratory factor 1 (NRF1), sequestosome-1 (P62), sirtuin 3 (SIRT3) and peroxisome proliferator-activated receptor gamma coactivator 1-alpha (PGC1α) were assessed by Western Blotting.

Liver samples (100 mg) were homogenised in 1 mL of cellular PBS (pH 7.4) containing protease inhibitors (100 mM phenylmethylsulphonyl fluoride and 100 mM iodoacetamide). The homogenates were then centrifuged (800× *g*, 10 min, 4 °C) and the protein concentration of supernatants was measured using the Bradford method [26] with BSA as a standard.

For immunoblot analysis, 60 μg of protein was denaturalised at 95 °C for 3 min in Laemmli buffer and loaded into either 4–15% (DGAT2 and FATP2) or 4–20% (ACC, pACC, TFAM, AQP9, FAS, CHREBP, NRF1, P62, SIRT3, PGC1α) Mini-PROTEAN TGX Precast Gels (BioRad, Hercules, CA, USA). The proteins were then transferred onto polyvinylidene difluoride (PVDF) membranes (IPVH00010, Millipore, Cork, Ireland) by electroblotting, and later blocked with 5% casein and 0.5% BSA PBS-tween buffer for 2 h at room temperature. After washing, membranes were incubated overnight at 4 °C with specific antibodies targeting DGAT2 (1:1000, Abcam, Cambridge, UK, ab59493), FATP2 (1:1000, Santa Cruz Biotechnology, Dallas, TX, USA, sc-161311), ACC (1:1000, Cell Signaling Technology, Danvers, MA, USA, 4190), pACC (1:1000, Cell Signaling Technology, Danvers, MA, USA, 3661), TFAM (1:1000, Santa Cruz Biotechnology, Dallas, TX, USA, sc-235588), AQP9 (1:1000, Santa Cruz Biotechnology, Dallas, TX, USA, sc-74409), FAS (1:1000, Abcam, Cambridge, UK, ab128870), CHREBP (1:1000, NovusBio, Centennial, CO, USA, NB400-135), NRF1 (1:1000, Abcam, Cambridge, UK, ab175932), P62 (1:1000, Abcam, Cambridge, UK, ab56416), SIRT3 (1:500, Santa Cruz Biotechnology Dallas, TX, USA, sc-365175), PGC1α (1:1000, Abcam, Cambridge, UK, ab54481) and α-tubulin (1:1000, Cell Signaling Technology, Danvers, MA, USA, 2125).

Following the washing step, proteins were detected through a 2 h incubation with secondary antibodies, including anti-rabbit (1:5000, Santa Cruz Biotechnology, Dallas, TX, USA, sc-2357), anti-goat (1:5000, Santa Cruz Biotechnology, Dallas, TX, USA, sc-2354) and anti-mouse (1:5000, Santa Cruz Biotechnology, Dallas, TX, USA, sc-516102) ones. The immunoreactive proteins were detected using the Forte Western HRP substrate (WBLUF0100, Millipore, MA, USA), and the blots were imaged by scanning with the ChemiDoc™MP Imaging System (Bio-Rad, Hercules, CA, USA). Specific bands were identified with a standard loading buffer (Precision Plus Protein standards dual colour, 161-0374, Bio-Rad, Hercules, CA, USA). α-Tubulin was used for normalisation reference.

### 2.7. Gene Expression by Real-Time PCR

Total RNA was extracted from 100 mg of liver using TRIzol^®^ (15596026, Invitrogen, Carlsbad, CA, USA) according to the manufacturer’s protocol. Genomic DNA was removed (DNase, Ambion, Foster City, CA, USA) and RNA concentration and quality (260/280 ratio) were determined using an RNA 6000 Nano Assay (Thermo Scientific, Wilmington, DE, USA). RNA samples were then treated with Recombinant DNase I (RNase-free) (2270A Takara Bio, Kusatsu, Japan). Total RNA (1.5 μg) was transcribed into complementary DNA (cDNA) using iScript cDNA Synthesis Kit (Bio-Rad, Hercules, CA, USA).

Gene expression levels of actin alpha 2 smooth muscle (*Acta2*), macrophage mannose receptor 1 (*CD206*), collagen type I alpha 1 chain (*Col1a1*), C-reactive protein (*Crp*), adhesion G protein-coupled receptor E1 (*F4*/*80*), interleukin 1 beta (*Il1b*), matrix metallopeptidase 9 (*Mmp9*), TIMP metallopeptidase inhibitor 1 (*Timp1*), and transforming growth factor beta 1 (*Tgfb1*) were determined by Real-Time PCR in the presence of SYBR Green Master Mix (Applied Biosystems, Foster City, CA, USA) using specific primer sequences (300 nM). β-Actin served as reference gene. Primer sequences are described in Table 1.

Monocyte chemoattractant protein 1 (*Mcp1*) and tumour necrosis factor α (*Tnfα*) were amplified using TaqMan probes (Mcp1: Accession Number NM_031530.1, Assay ID Rn00580555; Tnfα: Accession Number NM_012675.3, Assay ID Rn01525859) and the TaqMan Gene Expression Assay Mix (Applied Biosystems, Foster City, CA, USA). β-Actin (Accession Number NM_031144.3, Assay ID, Rn00667869) was used to normalise the expression levels.

All cDNA samples were amplified in an iCycler-MyiQ Real-Time PCR Detection System (Bio-Rad, Hercules, CA, USA). Results were expressed as fold changes calculated using the 2^−∆∆Ct^ method [27].

### 2.8. Parameters Related to Oxidative Stress in Liver

Lipid peroxidation was determined spectrophotometrically by measuring the formation of thiobarbituric acid reactive species (TBARS) using a commercial kit (TBARS Assay Kit 10009055, Cayman Chemical Company, Ann Arbor, MI, USA). The TBARS concentration in the samples was calculated by means of a standard curve obtained with malondialdehyde (MDA) and results were expressed as nM MDA·mg^−1^ tissue.

The total antioxidant capacity was assessed employing the commercial kit OxiSelect^TM^ Oxygen Radical Antioxidant Capacity (ORAC) activity assay (STA-345, Cell Biolabs, San Diego, CA, USA). Trolox solution was used to construct the calibration curve. The resulting ORAC values were expressed as μM Trolox equivalents·mg^−^^1^ protein.

The concentration of reduced glutathione (rGSH) in rat liver homogenates was colorimetrically assessed using a commercial kit (Glutathione Colorimetric Assay Kit, BioVision Incorporated K261, Milpitas, CA, USA), which relies on the glutathione recycling system in the presence of GSH and the DTNB fluorophore. The amount of GSH was calculated using a standard curve, and the results were expressed as μg rGHS·mg^−^^1^ protein.

The activity of glutathione peroxidase (GPx) was also assessed by measuring its H_2_O_2_ scavenging capacity using a colorimetric commercial kit (Glutathione Peroxidase Activity Colorimetric Assay Kit K762, BioVision Incorporated, Milpitas, CA, USA). The GPx levels in the samples were determined using a standard curve obtained with NADPH and results were expressed as mU GPx mg^−^^1^ protein.

Superoxide dismutase (SOD) was assessed spectrophotometrically using a commercial kit (Superoxide Dismutase Activity Assay Kit CS009, Sigma-Aldrich, St. Louis, MO, USA). This measurement involved quantifying the reduction in WST-1 formazan formation. SOD functions by quenching superoxide anions, leading to the conversion of WST-1 into WST-1 formazan. SOD activity was expressed as % inhibition rate.

Catalase activity was determined spectrophotometrically following the procedure described by Aebi [28], wherein the disappearance of H_2_O_2_ at 240 nm was monitored. Catalase activity was expressed as nmol·min^−^^1^·μg^−^^1^ protein.

In all instances, an Infinite 200Pro plate reader (Tecan, Männedorf, Zurich, Switzerland) was used.

### 2.9. Statistical Analysis

Results are presented as mean ± SEM. Statistical analysis was performed using SPSS 24.0 (SPSS, Chicago, IL, USA). The normal distribution of data was confirmed by the Shapiro–Wilk test. LC, OC and LGV groups on the one hand, and LC, OC, PF and HGV groups on the other hand were compared using a one-way ANOVA test, followed by the Newman–Keuls post hoc test. In the context of high-dose macroalga treatment, the inclusion of the pair-fed group facilitated the discernment of whether the alterations observed in the HGV group were attributable to the direct effect of the macroalga or were influenced by concomitant changes in food intake. Significance was assessed at the *p* < 0.05 level.

## 3. Results

### 3.1. Macroalga Composition

Dehydrated alga composition is summarised in Table 2. The analysis revealed that the major components of dried *Gracilaria vermiculophylla* are proteins (22.1%) and fibre (27.9%). Additionally, total polyphenol content accounted for 1180 mg/kg.

### 3.2. Body Weight, Energy Intake and Energy Efficiency Ratio

**Figure 1:**
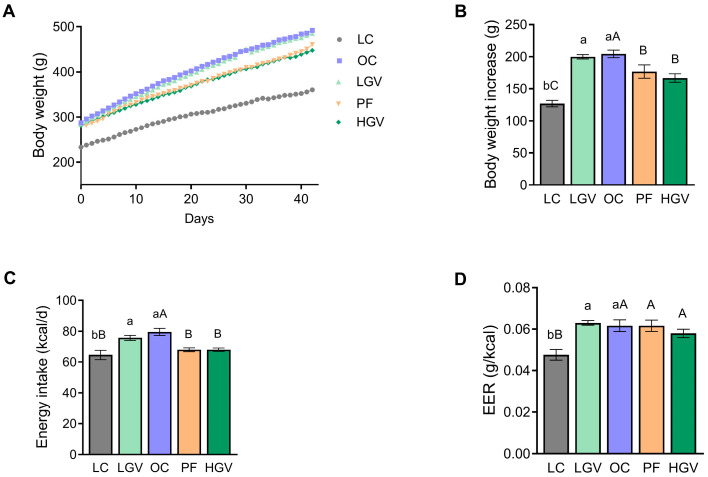
Body weight evolution curve (**A**), body weight increase (**B**), daily energy intake (**C**) and EER (**D**) of lean (LC) or obese Zucker rats fed with a standard diet (OC), a standard diet supplemented with 2.5% (LGV) or 5% (HGV) of *Gracilaria vermiculophylla* or a restricted amount of standard diet (PF). Values are presented as mean ± SEM. Lower cases represent differences among LC, OC and LGV groups, and upper cases represent differences among the LC, OC, PF and HGV groups. Values not sharing a common letter are significantly different (*p* < 0.05). EER: Energy Efficiency Ratio.

Figure 1A illustrates the trajectory of body weight across all groups throughout the entire experimental period. As expected, all obese Zucker rats showed higher body weight in comparison to their lean littermates during the intervention, as well as a greater body weight increase following the 6-week experimental period. The PF and HGV groups showed smaller body weight gain when compared to the OC group (Figure 1B).

The mean energy intake was higher in the OC group in comparison to the LC group. There were no differences between the OC and the LGV groups; however, in HGV, food intake was decreased, reaching values similar to those found in the LC counterpart (Figure 1C). In light of this circumstance, a pair-fed cohort (PF) was used elucidate whether changes induced by the elevated dosage of the alga were solely attributable to a reduction in food intake, or if additional direct metabolic effects were involved in the observed outcomes. When calculating the EER, a significant difference was found between lean and obese rats. All obese rats showed a greater EER than lean rats, but no significant differences were observed among groups of obese rats (Figure 1D).

### 3.3. Serum Biochemical Parameters

Serum glucose, insulin and NEFA levels were significantly higher in the OC group than in the LC cohort, suggesting insulin resistance (Table 3). This glycemic control alteration was confirmed by the values of both HOMA-IR and R-QUIKI indexes. LGV, HGV and PF groups reached physiological values of serum glucose. Regarding serum insulin levels, whereas the low dose of the alga did not modify this parameter, the high dose induced a significant reduction, which was similar to that observed in the PF group. When HOMA-IR was calculated, the treatment with the low dose of the alga did not modify this index, whereas in the high-dose and pair-fed groups, values were significantly decreased when compared with those of OC. Similar results were obtained in the case of R-QUICKY. Lastly, serum triglycerides were significantly higher in the OC rats than in their lean littermates. Among the experimental treatments, only the low dose of the alga induced a significant reduction (−26%), when compared to the OC (Table 3).

With regard to serum transaminases, the OC group exhibited higher ALT/GPT levels than the LC group. No changes were observed after treatment with the macroalga. In relation to AST/GOT, although the levels in the OC group did not differ from those in the LC counterpart, both groups supplemented with the macroalga demonstrated significantly higher values than the OC group. In the computation of the AST/ALT index, an indicative measure of hepatic disease, values were diminished in the OC group when compared with the LC cohort. Both groups receiving the macroalga along with PF exhibited higher values than the OC group. The ALP level, another parameter related to liver function, was increased in OC rats when compared to lean rats. Both groups receiving the macroalga showed increased levels in comparison to the OC group (Table 3).

Uric acid level augmented in OC rats when compared to the LC group. No significant differences were observed among the other experimental groups.

### 3.4. Liver Weight and Hepatic Index

**Figure 2:**
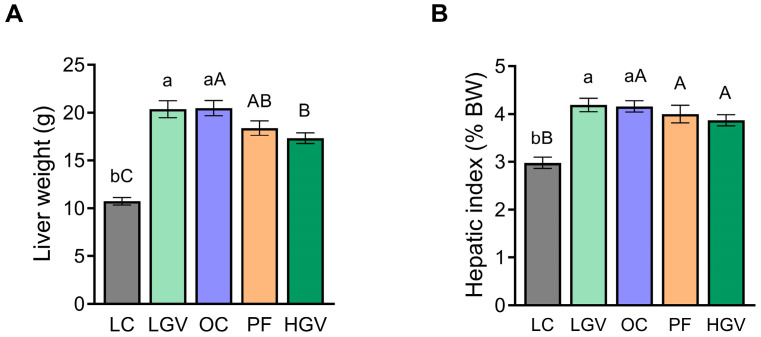
Liver weight (**A**) and hepatic index (expressed as percentage of body weight) (**B**) of lean (LC) or obese Zucker rats fed with a standard diet (OC), a standard diet supplemented with 2.5% (LGV) or 5% (HGV) of *Gracilaria vermiculophylla* or a restricted amount of standard diet (PF). Values are mean ± SEM. Lower cases represent differences among LC, OC and LGV groups, and upper cases represent differences among LC, OC, PF and HGV groups. Bars not sharing common letters are significantly different (*p* < 0.05). BW, body weight.

Liver weight in rats from the OC group was significantly higher than in lean rats. The supplementation with the high dose of the macroalga, but not with the low dose, resulted in a significant reduction in this parameter (Figure 2A). However, this did not translate into significant changes in the hepatic index (Figure 2B).

### 3.5. Hepatic Lipid Content

**Figure 3:**
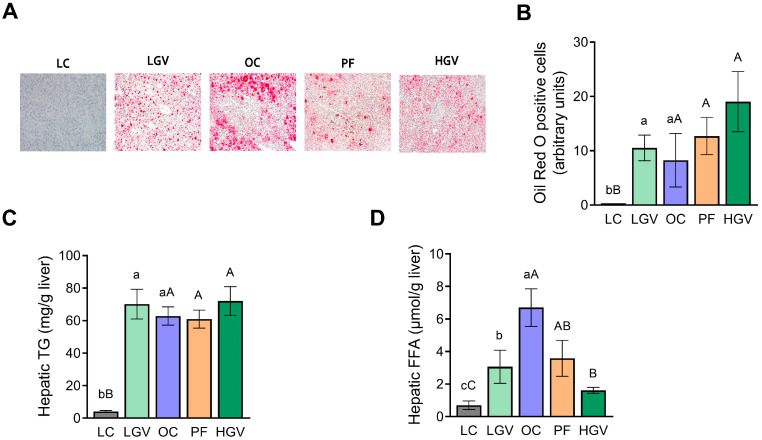
Representative Oil Red O staining (magnification ×20) (**A**), quantification of neutral lipids by Oil Red O (**B**), triglyceride (**C**) and NEFA (**D**) content in livers of lean (LC) or obese Zucker rats fed with a standard diet (OC), a standard diet supplemented with 2.5% (LGV) or 5% (HGV) of *Gracilaria vermiculophylla* or a restricted amount of standard diet (PF). Values are mean ± SEM. Lower cases represent differences among LC, OC and LGV groups, and upper cases represent differences among LC, OC, PF and HGV groups. Bars not sharing common letters are significantly different (*p* < 0.05). NEFA: non-esterified fatty acid, TG: triglyceride.

Genetically obese Zucker rats displayed a significant increase in hepatic total lipid content, triglycerides and NEFAs compared to the lean rats. *Gracilaria vermiculophylla* did not induce any changes concerning total lipid and triglyceride contents (Figure 3A–C); however, NEFA levels were significantly lower in the LGV and HGV groups than in the OC cohort (Figure 3D).

### 3.6. Activities of Enzymes and Expression of Proteins Involved in Fatty Acid and Triglyceride Synthesis

**Figure 4:**
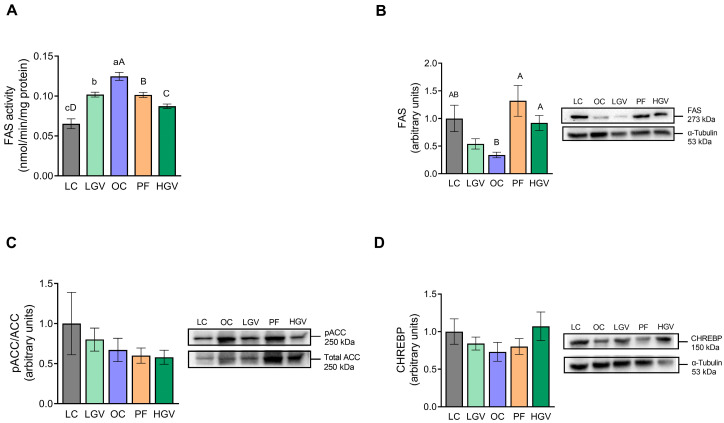
Activity of FAS (**A**), protein expression of FAS (**B**), phosphorylation ratio of ACC (**C**) and protein expression of CHREBP (**D**) in liver from lean (LC) or obese Zucker rats fed with a standard diet (OC), a standard diet supplemented with 2.5% (LGV) or 5% (HGV) of *Gracilaria vermiculophylla* or a restricted amount of standard diet (PF). Values are mean ± SEM. Lower cases represent differences among LC, OC and LGV groups, and upper cases represent differences among LC, OC, PF and HGV groups. Bars not sharing common letters are significantly different (*p* < 0.05). ACC: acetyl-CoA carboxylase, CHREBP: carbohydrate-responsive element-binding protein, FAS: fatty acid synthase.

Regarding de novo lipogenesis, FAS activity was higher in OC rats than in LC. This effect was reversed by both doses of the alga. In the case of the high dose, the reduction was significantly greater than that induced by food restriction in the PF group (Figure 4A). By contrast, FAS protein expression showed a tendency (*p* = 0.06) towards lower levels in the OC group when compared with LC. Although the low dose of the alga did not modify this parameter, the reduction observed in the OC group was completely reversed in the HGV and PF groups (Figure 4B). Concerning the pACC/totalACC ratio, recognised as an index of ACC activity (where a lower ratio indicates greater activation), no significant differences were observed among the experimental groups (Figure 4C). CHREBP expression, a transcription factor which regulates hepatic de novo lipogenesis, also remained unchanged among the experimental groups (Figure 4D).

**Figure 5:**
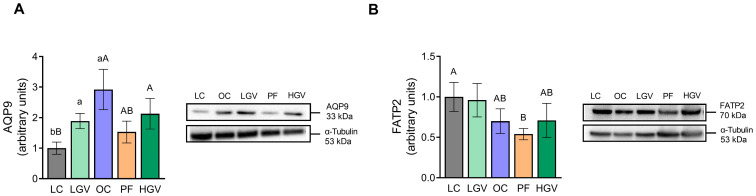
Protein expression of AQP9 (**A**) and FATP2 (**B**) in liver from lean (LC) or obese Zucker rats fed with a standard diet (OC), a standard diet supplemented with 2.5% (LGV) or 5% (HGV) of *Gracilaria vermiculophylla* or a restricted amount of standard diet (PF). Values are presented as mean ± SEM. Lower cases represent differences among LC, OC and LGV groups, and upper cases represent differences among LC, OC, PF and HGV groups. AQP9: aquaglyceroporin 9, FATP2: fatty acid transport protein 2.

Protein expression of AQP9 (Figure 5A) involved in glycerol uptake was increased in the OC group compared to the LC group, and dietary treatments did not induce significant changes. With regard to FATP2 responsible for fatty acid uptake, protein expression levels remained unaltered among the experimental groups (Figure 5B).

**Figure 6:**
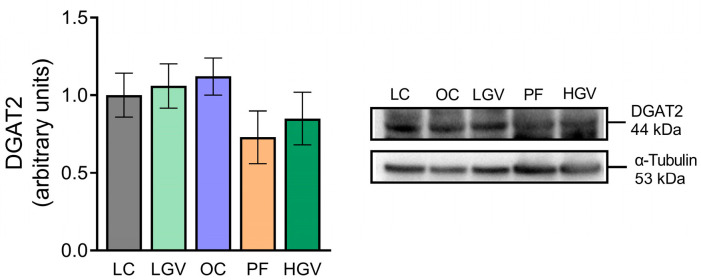
Protein expression of DAGT2 in liver from lean (LC) or obese Zucker rats fed with a standard diet (OC), a standard diet supplemented with 2.5% (LGV) or 5% (HGV) of *Gracilaria vermiculophylla* or a restricted amount of standard diet (PF). Values are mean ± SEM. DGAT2: diacylglycerol acyltransferase 2.

Furthermore, the expression of DGAT2, an enzyme responsible for triglyceride assembly that displayed no differences between obese and lean rats, was not modified by the dietary treatments (Figure 6).

### 3.7. Activities of Enzymes and Expression of Proteins Involved in Fatty Acid Oxidation and Mitochondriogenesis

**Figure 7:**
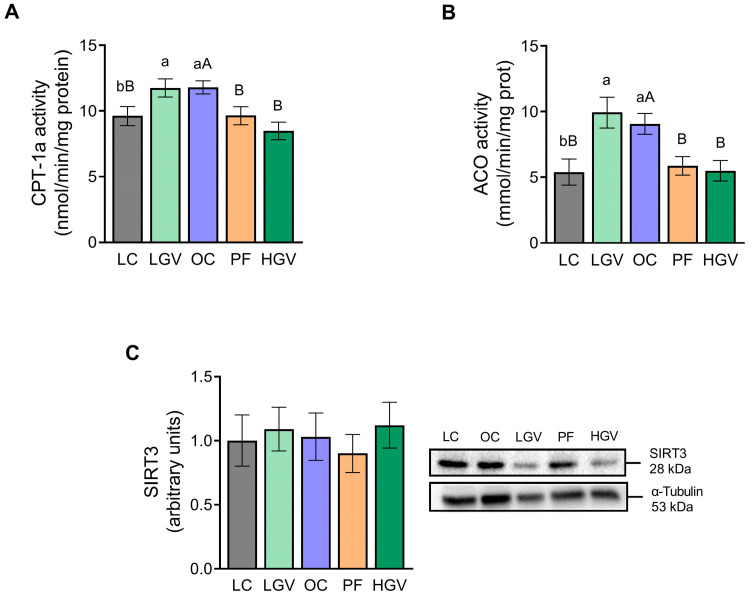
CPT-1a (**A**) and ACO (**B**) and protein expression of SIRT3 (**C**) in liver from lean (LC) or obese Zucker rats fed with a standard diet (OC), a standard diet supplemented with 2.5% (LGV) or 5% (HGV) of *Gracilaria vermiculophylla* or a restricted amount of standard diet (PF). Values are mean ± SEM. Lower cases represent differences among LC, OC and LGV groups, and upper cases represent differences among LC, OC, PF and HGV groups. Bars not sharing common letters are significantly different (*p* < 0.05). ACO: acyl-coenzyme A, CPT-1a: carnitine palmitoyltransferase-1a, SIRT3: sirtuin 3.

The activities of CPT-1a and ACO, key enzymes in mitochondrial and peroxisomal fatty acid oxidation, respectively, were measured. Both enzymes showed higher activity in the OC group in comparison to LC. Although supplementation with the low dose of the macroalga did not elicit any changes, both enzymes were decreased following supplementation with the high dose, reaching similar levels to those in lean rats, akin to the effects seen in the PF group (Figure 7A,B). Lastly, no changes in SIRT3 protein levels were observed among the experimental groups (Figure 7C).

**Figure 8:**
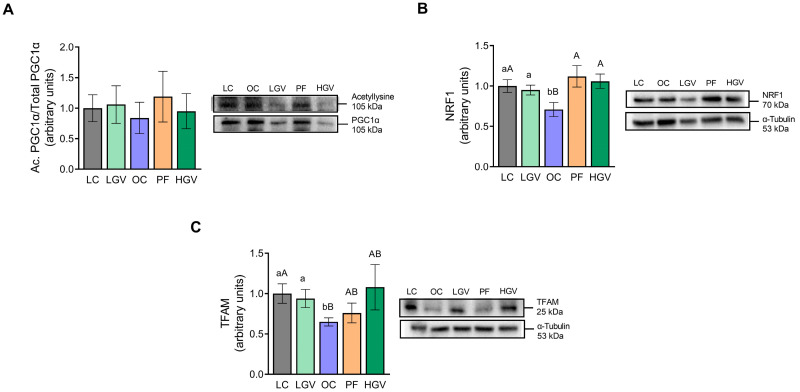
Acetylation ratio of PGC1α (**A**) and protein expression of NRF1 (**B**) and TFAM (**C**) in liver from lean (LC) or obese Zucker rats fed with a standard diet (OC), a standard diet supplemented with 2.5% (LGV) or 5% (HGV) of *Gracilaria vermiculophylla* or a restricted amount of standard diet (PF). Values are mean ± SEM. Lower cases represent differences among LC, OC and LGV groups, and upper cases represent differences among LC, OC, PF and HGV groups. Bars not sharing common letters are significantly different (*p* < 0.05). PGC1α: peroxisome proliferator-activated receptor gamma coactivator 1-alpha, NRF1: nuclear respiratory factor 1, TFAM: mitochondrial transcription factor A.

Concerning mitochondrial biogenesis, protein expressions of PGC1α, NRF1 and TFAM were measured. Acetylation levels of PGC1α remained unmodified among the experimental groups (Figure 8A). With regard to NRF1, rats in the OC group displayed a lower expression level than those in the LC group, and both doses of the macroalga were able to completely reverse this effect, reaching similar expression levels to those found in the LC group. In the PF cohort, NRF1 protein expression also increased compared to that in the OC group (Figure 8B). As for TFAM, reduced protein expression was observed in the OC group in comparison to LC. Rats receiving the low dose of the alga demonstrated increased protein expression, reaching the values yielded from the LC rats. However, the high dose induced no changes (Figure 8C).

### 3.8. Parameters Related to Triglyceride Secretion and Autophagy

**Figure 9:**
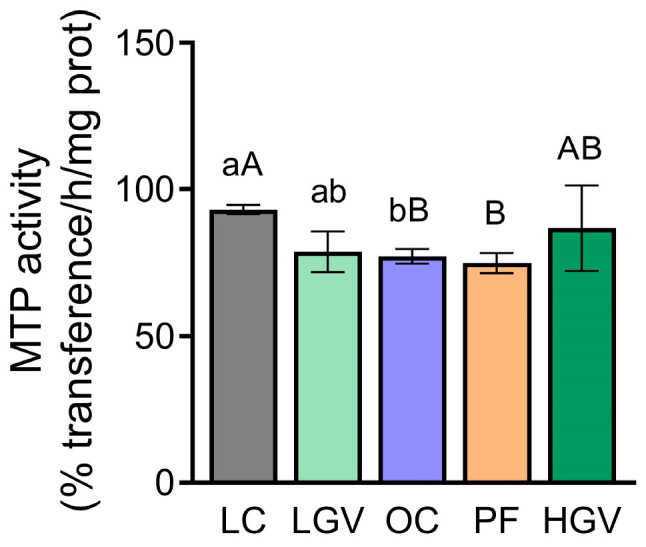
MTP activity in liver from lean (LC) or obese Zucker rats fed with a standard diet (OC), a standard diet supplemented with 2.5% (LGV) or 5% (HGV) of *Gracilaria vermiculophylla* or a restricted amount of standard diet (PF). Values are mean ± SEM. Lower cases represent differences among LC, OC and LGV groups, and upper cases represent differences among LC, OC, PF and HGV groups. Bars not sharing common letters are significantly different (*p* < 0.05). MTP: microsomal triglyceride transfer protein.

**Figure 10:**
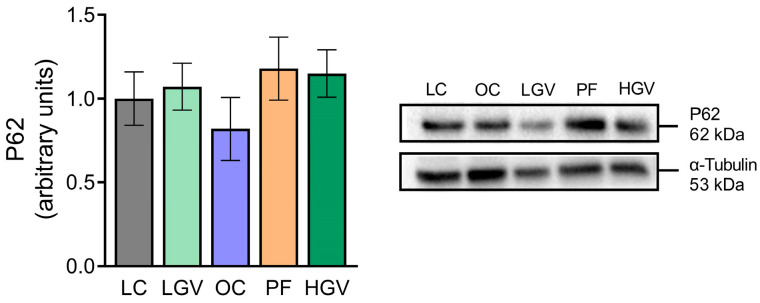
Protein expression of P62 in liver from lean (LC) or obese Zucker rats fed with a standard diet (OC), a standard diet supplemented with 2.5% (LGV) or 5% (HGV) of *Gracilaria vermiculophylla* or a restricted amount of standard diet (PF). Values are mean ± SEM. P62: sequestome-1.

No differences in the activity of MTP, an enzyme involved in triglyceride secretion from hepatocytes, were observed among the experimental groups (Figure 9). As for P62 protein levels considered to be a marker of the autophagic flux, a similar pattern without notable changes was observed among the experimental groups (Figure 10).

### 3.9. Hepatic Oxidative Stress Markers

**Figure 11:**
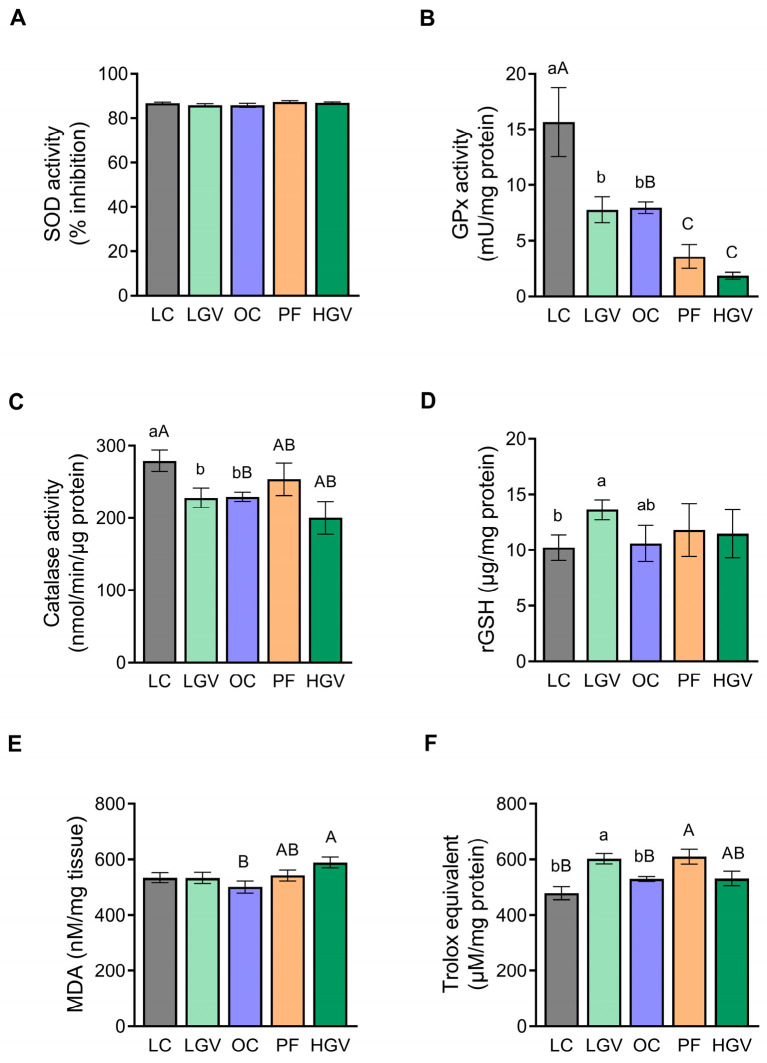
Activity of SOD (**A**), GPx (**B**) and catalase (**C**), and levels of rGSH (**D**), MDA (**E**) and Trolox equivalent (**F**) in liver from lean (LC) or obese Zucker rats fed with a standard diet (OC), a standard diet supplemented with 2.5% (LGV) or 5% (HGV) of *Gracilaria vermiculophylla* or a restricted amount of standard diet (PF). Values are mean ± SEM. Lower cases represent differences among LC, OC and LGV groups, and upper cases represent differences among LC, OC, PF and HGV groups. Bars not sharing common letters are significantly different (*p* < 0.05). GPx: glutathione peroxidase, rGSH: reduced glutathione, MDA: malondialdehyde, SOD: superoxide dismutase.

Regarding the activity of antioxidant enzymes, no differences were observed in SOD activity among the experimental groups (Figure 11A). By contrast, GPx activity was significantly lower in the OC group than in LC. The LGV group yielded no changes, while in the HGV group, GPx activity significantly decreased compared to the OC cohort. The PF rats similarly displayed reduced GPx activity in comparison with the OC group (Figure 11B). Concerning catalase, rats in the OC group exhibited a more reduced activity than those in the LC group, but no differences were observed among the other groups (Figure 11C). Regarding non-enzymatic antioxidant protection, no differences in rGSH levels were observed between the OC and LC groups. However, although not statistically significant (*p* = 0.1), values were increased by 30% in the LGV group when compared to OC (Figure 11D). Furthermore, MDA content, a marker of lipid peroxidation, was reduced in OC rats compared to their lean littermates. This reduction was reversed only in the HGV and PF groups (Figure 11E). Lastly, the total antioxidant capacity (Trolox equivalent) remained unaltered in the OC group compared to the LC counterpart. The administration of the low dose of the alga significantly increased this parameter in comparison to the OC group, but the high dose did not produce a similar effect. The PF group showed higher values than the OC cohort (Figure 11F).

### 3.10. Gene Expression of Inflammation-Related Markers in Liver

**Figure 12:**
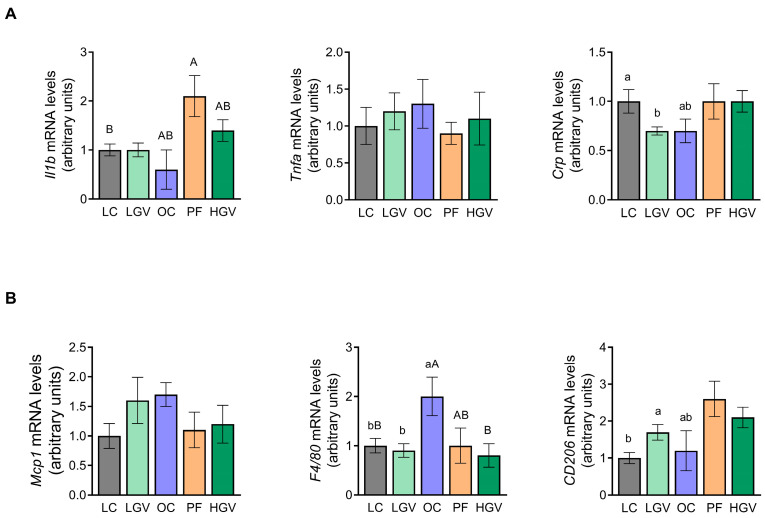
mRNA levels of inflammatory cytokines *Il1b*, *Tnfa* and *Crp* (**A**), and macrophage markers *Mcp1*, *F4*/*80* and *CD206* (**B**) in liver from lean (LC) or obese Zucker rats fed with a standard diet (OC), a standard diet supplemented with 2.5% (LGV) or 5% (HGV) of *Gracilaria vermiculophylla* or a restricted amount of standard diet (PF). Values are mean ± SEM. Lower cases represent differences among LC, OC and LGV groups, and upper cases represent differences among LC, OC, PF and HGV groups. Bars not sharing common letters are significantly different (*p* < 0.05). *CD206*: mannose receptor C, *Crp*: C-reactive protein, *F4*/*80*: adhesion G protein-coupled receptor E1, *Il1b*: interleukin 1b, *Mcp1*: monocyte chemoattractant protein 1, *Tnfa*: tumor necrosis factor α.

Figure 12A shows the gene expression of *Il1b*, *Tnfa* and *Crp* inflammatory cytokines in liver. In all instances, the gene expression of these cytokines remained unaltered in the OC group compared to the LC cohort. Additionally, none of the treatments induced alterations in these cytokines.

Regarding macrophage markers (Figure 12B), the mRNA level of *Mcp1*, a chemokine that promotes hepatic infiltration of macrophages, did not differ among the experimental groups. By contrast, the gene expression of *F4*/*80*, a marker of pro-inflammatory macrophages, was significantly up-regulated in the OC group in comparison to the LC group, while the supplementation with both doses of the alga decreased its expression to levels similar to those observed in the LC counterpart. The PF group showed intermediate values between the OC and HGV groups. Lastly, mRNA levels of *CD206*, a marker of anti-inflammatory macrophages, were not modified in the OC group in comparison to lean rats. Although no significant changes were observed among obese rats, gene expression was increased by 40%, 74% and 115% in LGV, HGV and PF, respectively, when compared to rats in the OC group.

### 3.11. Gene Expression of Fibrogenic Markers in Liver

**Figure 13:**
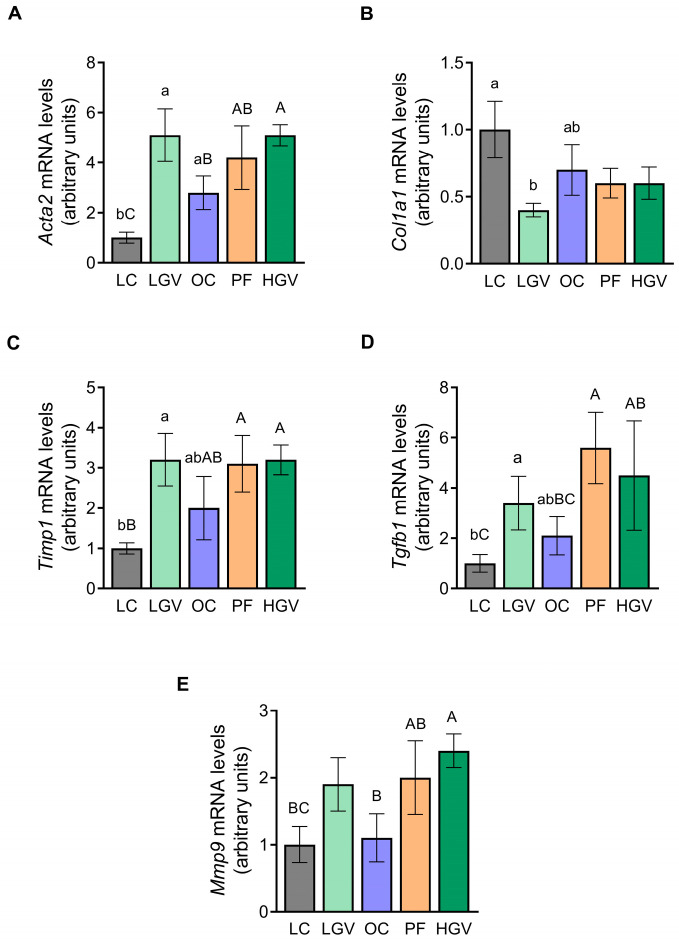
*Acta2* (**A**), *Col1a1* (**B**), *Timp1* (**C**), *Tgfb1* (**D**) and *Mmp9* (**E**) mRNA levels in liver from lean (LC) or obese Zucker rats fed with a standard diet (OC), a standard diet supplemented with 2.5% (LGV) or 5% (HGV) of *Gracilaria vermiculophylla* or a restricted amount of standard diet (PF). Values are mean ± SEM. Lower cases represent differences among LC, OC and LGV groups, and upper cases represent differences among LC, OC, PF and HGV groups. Bars not sharing common letters are significantly different (*p* < 0.05). *Acta2*: α-smooth muscle actin, *Col1a1*: collagen 1, *Mmp9*: matrix metallopeptidase 9, *Timp*: tissue inhibitor of matrix metalloproteases, *Tgfβ1*: transforming growth factor beta1.

*Acta2* mRNA levels were significantly higher in the OC group compared to the LC cohort. After the alga supplementation, while the low dose did not induce any change, rats in HGV showed increased *Acta2* gene expression when compared with OC. The PF group yielded intermediate values between the OC and HGV groups (Figure 13A). Regarding *Col1a1*, the OC group did not display any significant change in comparison to the LC counterpart. However, *Col1a1* expression decreased by 43% in LGV in comparison to OC, although statistical significance was not reached (Figure 13B). Regarding *Timp1*, only a slight tendency towards higher gene expression level was observed in the OC group (*p* = 0.07) compared with the LC cohort. After the alga supplementation, no changes were induced (Figure 13C). *Tgfb1* mRNA levels remained unmodified among the experimental groups (Figure 13D). Regarding matrix metallopeptidases, enzymes involved in extracellular matrix degradation, *Mmp9* gene expression was measured. No differences were observed in the OC group in comparison to LC, and after supplementation with the low dose, no significant differences were observed in comparison to OC rats, although an increase of 68% was noted. HGV rats exhibited significantly increased *Mmp9* mRNA levels compared to the OC group. The PF cohort showed intermediate values between OC and HGV groups (Figure 13E). Table S1 summarised the results obtained in the study.

## 4. Discussion

Marine macroalgae or seaweeds are garnering considerable attention due to their elevated nutritional value, particularly their rich content of protein, polyunsaturated fatty acids, fibres, minerals and vitamins [29]. Additionally, they represent a valuable yet underutilized source of novel compounds with antioxidant, anti-inflammatory, antiviral or anticancer activities, among other potential applications [11]. In fact, certain studies carried out in humans demonstrate that the consumption of the macroalgae can exert health benefits. For instance, it has been shown to improve the lipid profile [30], enhance insulin sensitivity [31] and glycemic control, and elevate levels of antioxidant enzymes [32].

With regard to hepatic steatosis, macroalgae consumption has shown to exert beneficial effects by decreasing hepatic fat deposition and other parameters related to fatty liver in experimental rat models [33,34,35] and mice [36,37,38]. Moreover, a recent study conducted in humans revealed a negative association between seaweed intake and NAFLD, particularly among non-obese subjects [39]

In the present study, we explored the impact of administering the red seaweed *Gracilaria vermiculophylla* for six weeks on hepatic steatosis in obese (*fa*/*fa*) Zucker rats. The obese Zucker rat serves as a widely used model to study liver diseases associated with obesity [40,41]. This model develops severe adiposity and impaired insulin sensitivity, fostering an elevated lipolytic flux originating from white adipose tissue, thus increasing plasmatic NEFA concentrations that reach the liver. Additionally, these rats exhibit heightened hepatic lipid synthesis and diminished oxidation, culminating in the development of liver steatosis [42,43].

Turning attention to glycemic control, glucose levels in the OC group were measured at 100 mg/dL, indicating a pre-diabetic stage. In contrast, glucose levels in the other experimental groups ranged from 74 to 78 mg/dL, signifying physiological values in these rats. These results suggest an improvement in insulin function in both alga-treated rats. The assessment of insulin levels revealed that in animals treated with the high dose of the macroalga, the improvement in insulin function could potentially be greater than that observed in animals treated with the low dose, as the serum concentration of this hormone was significantly reduced. The HOMA-IR index corroborated this suggestion. Taking into account that the obese rats exhibited increased serum NEFA levels, a metabolic condition often associated with impaired insulin sensitivity, the R-QUICKI index was also evaluated. The results were in accordance with those obtained from the assessment of HOMA-IR, indicating an improvement of insulin sensitivity following supplementation with the high dose of the alga. It is important to highlight that the positive effects induced by the high dose of the macroalga were attributed to the reduction in food intake resulting from this treatment, since the same effect was observed in the PF group.

Regarding serum triglycerides, it is noteworthy to mention that although no statistical differences were observed among obese rats, both OC and HGV groups exhibited triglyceride levels surpassing the threshold for hypertriglyceridemia (200 mg/dL). In contrast, the LGV and PF groups displayed values higher than physiological levels but below this threshold (150–199 mg/dL). For the LGV group, which showed a reduction of 26% in this parameter compared to the OC cohort, the triglyceride value reached almost physiological levels (151 mg/dL). This reduction could be explained, in part, by the effect of the macroalga on hepatic lipid metabolism, a topic discussed further.

Hepatic steatosis is characterised by increased accumulation of lipids in the liver, especially in the form of triglycerides. In a physiological status, only a relatively low quantity of triglycerides (less than 5%) is stored in cytoplasmic lipid droplets [44]. However, in obese Zucker rats, hepatic triglyceride content is highly elevated [41]. In the present experiment, as anticipated, obese rats exhibited higher total lipid content than lean Zucker rats, featuring increased amounts of triglycerides. These elevated concentrations contribute, at least in part, to the greater liver weight and hepatic index observed in obese rats. The heightened levels of triglyceride and NEFA in serum, could, in part, explain the increased lipid content observed in the livers of obese rats. Following supplementation with *Gracilaria vermiculophylla*, no changes in liver weight, hepatic lipid or triglyceride content were observed, suggesting that the macroalga supplementation was not effective in reducing hepatic steatosis.

To the best of our knowledge, this is the first study dedicated to investigating the effect of administering whole *Gracilaria vermiculophylla* on NAFLD. However, there is a study conducted in rats using 5% and 10% *Gracilaria changii* alga powder consumed with a high cholesterol/fat diet for eight weeks, where the treated groups exhibited ameliorated hepatic steatosis [45]. One of the doses used by Chan et al. aligns with that in our study; however, the discrepancies between both studies could primarily stem from the use of different *Gracilaria* species, which may lead to variations in the composition of bioactive compounds [11,46]. In addition, other differences in the experimental design may contribute to these discrepancies. Chan et al. employed a dietetic model of steatosis with an experimental period of eight weeks, while in the present study, a genetic model of steatosis and a shorter experimental period (six weeks) were used. Considering that approximately two weeks of a rat’s live equate to one human year [47], a two-week disparity between the two protocols could indeed yield different outcomes. Based on these findings, it could be hypothesised that the alga may require longer treatment periods to modify hepatic lipid content, and different experimental models may respond divergently to the treatment.

Further studies on *Gracilaria* sp. have been documented, opting for the use of alga extracts, specific components or fractions rather than the entire alga. In this line, a study conducted in mice fed with a high fat diet observed that the administration of a sulphated polysaccharide from *Gracilaria lemaneiformis* was effective in reducing hepatic lipid deposition [14,15]. In another study, the administration of *Gracilaria chorda* subcritical water extract in obese C57BL/6J mice ameliorated hepatic lipid accumulation [13]. The discrepancies between the present study and those reported in the literature may arise from the higher concentration of the mentioned bioactive compounds in the fractions and extracts compared with the whole alga. In addition, mice generally exhibit greater responsiveness to treatments than rats [48].

In hepatic steatosis, besides triglycerides, other important lipid species include NEFAs, which contribute even more significantly to hepatocyte injury, primarily through the formation of various toxic lipid species such as ceramides, diacylglycerols and lysophosphatidylcholine. These metabolites favour mitochondrial dysfunction, resulting in an elevated production of reactive oxygen species (ROS) [49]. In the present study, supplementation with *Gracilaria vermiculophylla* led to a reduced hepatic NEFA content. Notably, in the case of the highest dose of the alga, the observed reduction was partially due to the decrease in food intake. This is evident as the PF group displayed intermediate values between the OC and HGV groups, with no significant differences between the PF and HGV cohorts.

In order to explain the reduction observed in NEFAs, the main enzymes involved in de novo lipogenesis were studied. The phosphorylation ratio of ACC was diminished by 33% in the OC group compared to their lean counterparts, indicating major ACC activation levels compared to those in lean rats. This effect, together with the enhanced FAS activity observed in obese rats, suggests an increase in de novo lipogenesis that may contribute to the hepatic pool of NEFAs. Supplementation with *Gracilaria vermiculophylla* reduced the increased FAS activity. Taking into account that the low dose of the macroalga did not modify FAS protein expression and, despite the increase after supplementation with the high dose, FAS activity decreased in both cases, it appears that this change in FAS activity occurred at the post-translational level [50]. Thus, it can be proposed that *Gracilaria vermiculophylla* supplementation lowered hepatic NEFA content, in part, due to the decrease in de novo lipogenesis.

Additionally, we investigated whether changes in fatty acid oxidation were also involved either in the reduction of hepatic NEFAs or in serum triglycerides. This process occurs via β-oxidation and takes place in mitochondria and, to a lesser extent, in peroxisomes. The rate-limiting step for mitochondrial fatty acid oxidation is catalysed by CPT-1, and in peroxisomes, ACO catalyses the first reaction of β-oxidation. We analysed the activity of these two enzymes in the mitochondrial/peroxisomal fraction of hepatocytes, and subsequent to macroalga supplementation, only the high dose demonstrated the capacity to diminish the values. Nevertheless, this change was due to the reduction in food intake, as similar changes were observed in the PF group. Moreover, we noted an increase in the mitochondriogenesis with the low dose of the alga, as indicated by the increase in NRF1 and TFAM gene expression. This effect was less pronounced in the case of the low dose of the alga, as its supplementation only resulted in a significant increase in NRF1. On the other hand, the boost observed in the pair-fed rats may suggest that a lower food intake (a slight energy restriction) could potentially restore NRF1 levels in non-treated obese rats. Additionally, the supplementation with the highest dose does not seem to independently affect mitochondrial biogenesis. These results may suggest that, in the case of the low dose, the alga can enhance fatty acid oxidation by increasing the number of mitochondria. Nonetheless, in the case of the high dose, it does not exert this direct effect. Taken together, these findings, along with the decrease in de novo lipogenesis, could explain the reduction observed in hepatic NEFA content as well as serum triglycerides (−26%).

Oxidative stress plays a central role in the progression of NAFLD towards NASH, since it triggers inflammatory and fibrogenic pathways [7]. Zucker rats fed with a standard diet exhibit increased oxidative stress biomarkers at 14 weeks [51]. This timeframe aligns with the age of the rats in the present study upon completion of the experimental period. Furthermore, certain algae are known to contain antioxidant molecules [52] and it has been demonstrated that specific compounds, such as N-acetylcysteine, possess the potential to improve oxidative stress independently of their ability to reduce lipid accumulation in the liver [53]. On the one hand, the decrease in hepatic NEFA may imply a reduction in lipid peroxidation [8], and on the other hand, the increase in fatty acid oxidation can lead to elevated ROS production [54]. Given this scenario, an investigation into oxidative stress was conducted.

Based on our findings, the activity of the antioxidant enzymes catalase and GPx was markedly reduced in obese rats. However, supplementation with *Gracilaria vermiculophylla* failed to improve this alteration; instead, the high dose of the alga induced an additional reduction. This effect was not a direct consequence of the alga itself, but rather associated with the reduction in food intake induced by this treatment. This is supported by the observation that rats from the PF group exhibited the same effect. Regarding the non-enzymatic antioxidant defense, rGSH was slightly increased (*p* = 0.1) by the low dose of the alga. In relation to the high dose, surprisingly, it led to an increase in lipid peroxidation, as evidenced by the enhancement of MDA, a phenomenon that appears to be unrelated to the reduction in food intake. Lastly, the total antioxidant capacity (Trolox) was increased by the low dose of the alga. Collectively, these results suggest that, under our experimental conditions, the low dose of the macroalga can exert some beneficial effects regarding oxidative stress, whereas the highest one seems to promote lipid peroxidation.

These effects are not in line with other studies in the literature. Chan et al. reported that rats fed with a high cholesterol/fat diet enriched with 5% and 10% *Gracilaria changii* whole algae powder for 8 weeks displayed improved lipid peroxidation (MDA levels) as well as antioxidant enzymes (catalase and GPx). However, SOD activity was only increased after supplementing with 10%, and no changes were observed with 5% [55]. In another study where oligosaccharides from *Gracilaria lemaneiformis* were used, the authors observed antioxidant activity in a mice model of alcohol-induced liver damage. This was evident through a reduction in MDA levels, a boost in GSH levels and enhanced SOD activity [56]. In an additional study, *Gracilaria birdiae* extract was found to increase hepatic catalase activity and total antioxidant capacity, with no discernible impact on SOD and GSH [57]. It is important to note that the observed discrepancies may be attributed to the significant differences in experimental designs between those studies and the present research.

It is well-established that Zucker rats do not spontaneously develop NASH unless exposed to an external stimulus such as a high-fat diet [58]. Therefore, in the present study, rats were fed a standard diet, and consequently, the onset of NASH was not anticipated. Nevertheless, the analysis of potential changes in early biomarkers of inflammation and fibrosis could be of interest. Regarding inflammation, although mRNA levels of pro-inflammatory cytokines did not differ among the experimental groups, the reduction in *F4*/*80*, a marker of pro-inflammatory (M1) Kupffer cells observed in rats receiving *Gracilaria vermiculophylla*, suggests an anti-inflammatory effect of this macroalga.

As far as fibrosis is concerned, in general terms, the alga treatment did not have any significant effects on the studied markers (*Col1a1*, *Timp1*, *Tfgb1*). Nevertheless, our results showed an increase in hepatic *Mmp9* mRNA levels after supplementing rats with the macroalga. It is known that the activation of MMP-9 degrades collagen in liver, and for that reason, it is sometimes augmented in hepatic fibrosis [59], probably as a compensatory mechanism. However, in our study, since livers did not exhibit fibrosis or an increase in fibrotic markers, *Mmp9* is probably acting as a protective factor to prevent the potential development of fibrosis.

Regarding the limitations of this study, it is important to note that the precise composition and bioactive compounds found in *Gracilaria vermiculophylla* are not determined. This lack of knowledge hinders the identification of the specific component or components responsible for the observed biological effects. Nevertheless, it should be emphasized that, even in the case of having this information, the bioactive compounds should be commercially available in order to check their individual activities. Otherwise, they should be synthesized or extracted by a group of researchers with good expertise in this field. On the other hand, bioactive compound bioavailability should be determined, but the experimental conditions of the present study are not appropriate to investigate this issue. Thus, the fasting period used in the present design is not suitable. Moreover, since blood samples should be collected several times within a specific interval, the stress induced by this sample obtaining would likely alter some of the parameters evaluated in the present study. Consequently, an independent study carried out with a different experimental design would be necessary. Another limitation is that the effects of the alga on inflammation and fibrosis cannot be assessed in this study, since the obese Zucker rat does not spontaneously develop steatohepatitis. For this purpose, feeding these rats a high-fat diet would be required. Furthermore, a dose response study was not carried out in this work; instead, only two doses were used. This restricts our understanding of the effectiveness of this alga within a specific dosage range and, consequently, the fact that doses within that range could potentially be more effective than the low dose (2.5% in the diet) cannot be ruled out. Moreover, it is worth noting that this study solely focused on male rats. Therefore, it remains uncertain whether the observed effects would be the same, lesser or even greater in females. This could be possible since there is evidence pointing to the fact that the development of steatosis occurs differently in males and females [60]. Another important aspect is that it remains unknown why the consumption of a high dose of the alga resulted in a decrease in food intake. It could be due to a decrease in appetite, for instance, after modification of neuropeptides or simply because animals dislike the taste of seaweed; this requires further research. Additionally, the potential involvement of gut microbiota in the observed effects was not addressed. Finally, since this is a preclinical study, it is important to be aware that the translation of the observed effects to humans is not guaranteed. Consequently, further research is needed to evaluate the efficacy of the alga in clinical trials.

## 5. Conclusions

In summary, the findings reported in the present study indicate, for the first time, that while *Gracilaria vermiculophylla* may not reduce lipid and triglyceride accumulation in the liver, it does confer some beneficial effects, such as reductions in hepatic NEFAs, oxidative stress and inflammation makers. These effects are dose-dependent, and in the range used in the present study, only the low dose demonstrated effectiveness. Such effects probably make this alga a promising functional food that may have significant implications in the prevention of the appearance of complications associated with fatty liver.

Regarding future research, efforts should be made to identify the bioactive compounds responsible for the alga effects, as well as to determine their bioavailability. Further studies are also needed to determine the best choice for supplementation: some individual compounds, a combination of them or the whole alga, as in the present study.

## Figures and Tables

**Table 1:** Primer sequences for quantitative Real-Time PCR amplification.

Gene	Accession Number	Sense Primer 5′-3′	Antisense Primer 5′-3′
*Acta2*	NM_031004.2	GCC GAG ATC TCA CCG ACT AC	GTC CAG AGC GAC ATA GCA CA
*Actb*	NM_031144.3	CCC GCG AGT ACA ACC TTC T	CGT CAT CCA TGG CGA ACT
*CD206*	NM_001106123.2	ACT GCG TGG TGA TGA AAG G	TAA CCC AGT GGT TGC TCA CA
*Col1a1*	NM_053304.1	TCC TGG CAA GAA CGG AGA T	CAG GAG GTC CAC GCT CAC
*Crp*	NM_017096.4	TGT CTC TAT GCC CAC GCT GAT G	GGC CCA CCT ACT GCA ATA CTA AAC
*F4*/*80*	NM_001007557.2	CTC TTC CTG ATG GTG AGA AAC C	CCC ATG GAT GTA CAG TAG CAG A
*Il1b*	NM_031512.2	TGT GAT GAA AGA CGG CAC AC	CTT CTT CTT TGG GTA TTG TTT GG
*Mmp9*	NM_031055.2	AGC CGA CGT CAC TGT AAC TG	CCA GGA AGA CGA AGG GGA AG
*Timp1*	NM_053819.1	CAG CAA AAG GCC TTC GTA AA	TGG CTG AAC AGG GAA ACA CT
*Tgfb1*	NM_021578.2	CCT GGA AAG GGC TCA ACA C	TGC CGT ACA CAG CAG TTC TT

*Acta2*, Actin alpha 2 smooth muscle; *Actb*, beta actin; *CD206*, macrophage mannose receptor 1; *Col1a1*, collagen type I alpha 1 chain; *Crp*, C-reactive protein; *F4*/*80*, adhesion G protein-coupled receptor E1; *Il-1b*, interleukin 1 beta; *Mmp9*, matrix metallopeptidase 9; *Timp1*, TIMP metallopeptidase inhibitor 1; *Tgf b1*, transforming growth factor beta 1.

**Table 2:** Composition of *Gracilaria vermiculophylla*.

	*Gracilaria vermiculophylla*
Energy (kcal/100 g)	182
Total lipids (g/100 g)	1.40
Saturated fatty acids (g/100 g)	0.98
Total carbohydrates (g/100 g)	6.40
Simple carbohydrates (g/100 g)	<0.50
Proteins (g/100 g)	22.10
Fibre (g/100 g)	27.90
Ashes (g/100 g)	28.8
Moisture (g/100 g)	13.4

**Table 3:** Serum biochemical variables of rats from different experimental groups.

	LC	LGV	OC	PF	HGV
Glucose (mg/dL)	75 ± 7 ^bB^	76 ± 12 ^ab^	100 ± 8 ^aA^	74 ± 4 ^B^	78 ± 9 ^AB^
Insulin (ng/mL)	0.8 ± 0.1 ^bC^	25.3 ± 4.1 ^a^	26.6 ± 1.5 ^aA^	17.5 ± 3.2 ^B^	15.8 ± 2.4 ^B^
HOMA-IR	3.5 ± 0.7 ^bC^	119.5 ± 28.2 ^a^	174.2 ± 13.3 ^aA^	70.8 ± 13.6 ^B^	77.1 ± 15.4 ^B^
TG (mg/dL)	26 ± 3 ^bB^	151 ± 27 ^a^	206 ± 50 ^aA^	199 ± 33 ^A^	219 ± 50 ^A^
NEFA (mg/dL)	8.7 ± 0.9 ^bB^	35.4 ± 4.4 ^a^	29.3 ± 2.0 ^aA^	32.0 ± 3.6 ^A^	37.0 ± 6.5 ^A^
R-QUICKI	0.38 ± 0.02 ^aB^	0.29 ± 0.01 ^b^	0.28 ± 0.01 ^bC^	0.31 ± 0.01 ^A^	0.31 ± 0.01 ^A^
ALT/GPT (U/L)	56 ± 2 ^bB^	119 ± 11 ^a^	110 ± 12 ^aA^	98 ± 7 ^A^	119 ± 13 ^A^
AST/GOT (U/L)	142 ± 8 ^bB^	190 ± 9 ^a^	144 ± 11 ^bB^	166 ± 11 ^AB^	199 ± 16 ^A^
AST/ALT	2.6 ± 0.1 ^aA^	1.8 ± 0.1 ^b^	1.4 ± 0.1 ^cC^	1.7 ± 0.1 ^B^	1.6 ± 0.1 ^B^
ALP (U/L)	278 ± 6 ^cC^	515 ± 24 ^a^	388 ± 15 ^bB^	400 ± 15.1 ^B^	542 ± 36 ^A^
Uric acid (mg/mL)	1.6 ± 0.1 ^bB^	5.0 ± 0.8 ^a^	4.3 ± 0.4 ^aA^	3.6 ± 0.5 ^A^	6.4 ± 1.3 ^A^

Values are presented as mean ± SEM. Lower cases represent ANOVA between LC, OC and LGV groups, and upper cases represent ANOVA between LC, OC, PF and HGV groups. Values not sharing a common letter are significantly different (*p* < 0.05). Experimental groups: HGV, *Gracilaria vermiculophylla* 5%; LC, lean control; LGV, *Gracilaria vermiculophylla* 2.5%; OC, obese control; PF, pair-fed. ALP: alkaline phosphatase, ALT/GPT: alanine aminotransferase, AST/GOT: aspartate aminotransferase, HOMA-IR: homeostatic model assessment for insulin resistance, NEFA: non-esterified fatty acid, R-QUICKI: revised quantitative insulin sensitivity check index, TG: triglyceride.

## Data Availability

Data are contained within the article and Supplementary Materials.

## Supplementary Materials

The following supporting information can be downloaded at: https://www.mdpi.com/article/10.3390/antiox13030369/s1, Table S1: Summary table of the results obtained in the present study.
