# Supplementary material for: Effect of Gracilaria vermiculophylla Macroalga on Non-Alcoholic Fatty Liver Disease in Obese Rats
Source: Antioxidants (Basel). 2024 Mar 18;13(3):369. doi: 10.3390/antiox13030369 (PMC10968416; doi:10.3390/antiox13030369)
Supplement: Supplementary file 1 [file antioxidants-13-00369-s001.zip › antioxidants-2864413-supplementary.pdf]

Table S1. Summary table of the results obtained in the present study.

	(OC, LGV, PF and HGV) vs LC							
	**OC**	**LGV**	**PF**	**HGV**	**LGV vs OC**	**HGV vs OC**	**PF vs OC**	**HGV vs PF**
Energy intake and somatometric parameters								
Energy intake	↑	↑	NS	NS	NS	↓	↓	NS
Energy efficiency ratio	↑	↑	↑	↑	NS	NS	NS	NS
Body weight increase	↑	↑	↑	↑	NS	↓	↓	NS
Liver weight	↑	↑	↑	↑	NS	↓	NS	NS
Hepatic index	↑	↑	↑	↑	NS	NS	NS	NS
Serum parameters								
Glucose	↑	NS	NS	NS	NS	NS (↓)	↓	NS
Insulin	↑	↑	↑	↑	NS	↓	↓	NS
HOMA-IR	↑	↑	↑	↑	NS	↓	↓	NS
Triglycerides	↑	↑	↑	↑	NS	NS	NS	NS
NEFAs	↑	↑	↑	↑	NS	NS	NS	NS
R-QUICKI	↓	↓	↓	↓	NS	↑	↑	NS
ALT/GPT	↑	↑	↑	↑	NS	NS	NS	NS
AST/GOT	NS	↑	NS (↑)	↑	↑	↑	NS	NS
AST/ALT	↓	↓	↓	↓	↑	↑	↑	NS
ALP	↑	↑	↑	↑	↑	↑	NS	↑
Uric acid	↑	↑	↑	↑	NS	NS	NS	NS (↑)
Hepatic composition								
Total lipids	↑	↑	↑	↑	NS	NS	NS	NS
Triglycerides	↑	↑	↑	↑	NS	NS	NS	NS
NEFAs	↑	↑	↑	↑	↓	↓	NS (↓)	NS
Hepatic lipid metabolism								
FAS activity	↑	↑	↑	↑	↓	↓	↓	↓
FAS protein expression	NS (↓)	NS (↓)	NS	NS	NS	↑	↑	NS
pACC/total ACC	NS	NS	NS	NS	NS	NS	NS	NS
CHREBP	NS	NS	NS	NS	NS	NS	NS	NS
AQP9	↑	↑	NS	↑	NS	NS	NS (↓)	NS
FATP2	NS	NS	↓	NS	NS	NS	NS	NS
DGAT2	NS	NS	NS	NS	NS	NS	NS (↓)	NS

CPT-1a	↑	↑	NS	NS	NS	↓	↓	NS
ACO	↑	↑	NS	NS	NS	↓	↓	NS
SIRT3	NS	NS	NS	NS	NS	NS	NS	NS
Ac. PGC1α/total PGC1α	NS	NS	NS	NS	NS	NS	NS	NS
NRF1	↓	NS	NS	NS	↑	↑	↑	NS
TFAM	↓	NS	NS	NS	↑	NS	NS	NS
MTP	↓	NS (↓)	↓	NS	NS	NS	NS	NS
P62	NS	NS	NS	NS	NS	NS	NS	NS
Oxidative stress related markers								
SOD	NS	NS	NS	NS	NS	NS	NS	NS
GPx	↓	↓	↓	↓	NS	↓	↓	NS
Catalase	↓	↓	NS	NS	NS	NS	NS	NS
rGSH	NS	↑	NS	NS	NS (↑)	NS	NS	NS
MDA	↓	NS	NS	↑	NS (↑)	↑	NS	↑
Trolox	NS (↑)	↑	↑	NS	↑	NS	↑	NS (↓)
Inflammation related markers								
Il1b	NS	NS	↑	NS	NS	NS	NS (↑)	NS
Tnfa	NS	NS	NS	NS	NS	NS	NS	NS
Crp	NS (↓)	↓	NS	NS	NS	NS	NS	NS
Mcp1	NS (↑)	NS	NS	NS	NS	NS	NS	NS
F4/80	↑	NS	NS	NS	↓	↓	NS	NS
CD206	NS	↑	↑	↑	NS	NS	NS	NS
Fibrosis related markers								
Acta2	↑	↑	↑	↑	NS (↑)	↑	NS	NS
Col1a1	NS	↓	NS (↓)	NS	NS	NS	NS	NS
Timp1	NS (↑)	↑	↑	↑	NS	NS	NS	NS
Tfgb1	NS	↑	↑	↑	NS	NS	↑	NS
Mmp9	NS	NS (↑)	NS (↑)	↑	NS	↑	NS	NS

ACC: acetyl-CoA carboxylase, ACO: acyl-coenzyme A, Acta2: α-smooth muscle actin, ALP: alkaline phosphatase, ALT/GPT: alanine aminotransferase, AQP9: aquaglyceroporin 9, AST/GOT: aspartate aminotransferase, CD206: mannose receptor C, CHREBP: carbohydrate-responsive element-binding protein, Col1a1: collagen 1, CPT-1a: carnitine palmitoyltransferase-1a, Crp: C-reactive protein, DGAT2: diacylglycerol acyltransferase 2, F4/80: adhesion G protein-coupled receptor E1, FAS: fatty acid synthase, FATP2: fatty acid transport protein 2, GPx: glutathione peroxidase, HOMA-IR: homeostatic model assessment for insulin resistance, Il1b: interleukin 1b, Mcp1: monocyte chemoattractant protein 1, MDA: malondialdehyde, Mmp9: matrix metallopeptidase 9, MTP: microsomal triglyceride transfer protein, NEFA: non-esterified fatty

acid, NRF1: nuclear respiratory factor 1, P62: sequestome-1, PGC1α: peroxisome proliferator-activated receptor gamma coactivator 1-alpha, rGSH: reduced glutathione, R-QUICKI: revised quantitative insulin sensitivity check index, SIRT3: sirtuin 3, SOD: superoxide dismutase, TFAM: mitochondrial transcription factor A, Tgfβ1: transforming growth factor beta1, Timp: tissue inhibitor of matrix metalloproteases, Tnfa: tumor necrosis factor α. NS: non-significant, ↑: increase, ↓: decrease, NS (↑): tendency towards increased values, NS (↓): tendency towards decreased values. Experimental groups: HGV, *Gracilaria vermiculophylla* 5 %; LC, lean control; LGV, *Gracilaria vermiculophylla* 2.5 %; OC, obese control; PF, pair-fed.
